# Diet & Nutrition: Expert Panel Weighs In on Global Cancer Control

**DOI:** 10.1289/ehp.116-a22b

**Published:** 2008-01

**Authors:** M. Nathaniel Mead

Cancer originates in genetic mutations, but diet, activity level, and other lifestyle factors play a critical role in determining whether these mutations occur, making cancer a largely preventable disease. This is among the main conclusions of *Food, Nutrition, Physical Activity, and the Prevention of Cancer: A Global Perspective*, released in November 2007 by the World Cancer Research Fund (WCRF) International and the American Institute for Cancer Research (AICR).

WCRF International/AICR commissioned 9 systematic literature review (SLR) teams comprising 22 panelists to summarize the literature on nutrition, physical activity, and cancer. The teams examined 7,000 articles, reviews, and meta-analyses in all languages. Team findings went to an international panel that synthesized information for many different cancers to come up with the report’s main recommendations.

The report lays out a cogent groundwork for understanding how diet, exercise, and other lifestyle factors affect cancer risk, as well as how to use this information for more effective cancer prevention on a global scale. The public policy implications of the recommendations will be the subject of a further report to be published in late 2008.

The report concluded that about 40% of all cancers are linked to poor diet, physical inactivity, and suboptimal body weight. The panelists recommend maintaining a body mass index of between 21 and 23 (until now, the standard recommended range has been 18.5 to 24.5), exercising moderately, and limiting consumption of alcohol, high-fat foods, and refined carbohydrates such as sugary beverages. In addition to increasing vegetable intake, the report suggests replacing red meats and processed meats with poultry, fish, and eggs.

“The conclusions are really not surprising, but what did surprise me was the universality of the recommendations across so many countries,” says Tim Byers, a preventive medicine professor at the University of Colorado Cancer Center in Aurora. “I was surprised to find that nutritional factors that can lower cancer risk are shared widely across the world. . . . The importance of this report, therefore, is both that it is comprehensive and also that it is global.”

An earlier WCRF International/AICR report, published in 1997, stated that “cancer is principally caused by environmental factors, of which the most important are tobacco, diet and factors related to diet, including body mass and physical activity, and exposures in the workplace and elsewhere.” Although the panel still emphasizes the importance of not smoking and avoiding secondhand smoke, this report concentrated on the impacts of diet, activity, and weight.

Lawrence H. Kushi, associate director for etiology and prevention research at Kaiser Permanente in Oakland, California, and an SLR team co-leader, says this document is a substantial improvement over the first report in that it is more comprehensive. Moreover, he says the report is “groundbreaking [because] it established methodologies for conducting literature reviews and meta-analyses for observational epidemiologic data.”

Indeed, Kushi notes that the systematic review process relied primarily on analytical epidemiologic studies, such as prospective cohort studies and case–control studies. “Consistency of findings across these more rigorous study designs and [well-defined] populations form the bulk of the report and the basis of its recommendations,” he says.

Compelling evidence for the impact of environmental factors comes from studies describing changes in the rates of different cancers in populations that migrate from one country to another. For example, research in the 1980s showed that breast cancer incidence increased almost 3-fold in first-generation Japanese women who migrated to Hawaii, and up to 5-fold in the second generation. The migrant studies “prove that the main determinants of cancer patterns are environmental, and suggest that patterns of food, nutrition, and physical activity are important among these causes,” the report states.

“One of the project’s major strengths was the participation of many leaders in the field, from the development of the methodology, to the conduct of the SLRs, to issuing conclusions regarding the evidence and public health recommendations,” says SLR team leader Elisa Bandera, an epidemiologist at UMDNJ–Robert Wood Johnson Medical School and The Cancer Institute of New Jersey. “This is a very important resource for cancer prevention and public health education. It can guide researchers in identifying research gaps quickly, and ultimately should help guide public policy changes as well.” Moreover, she notes, WCRF International/AICR will update the evidence on its website as new findings emerge. For more information, go to http://www.dietandcancerreport.org/.

## Report Recommendations for Reducing Cancer Risk

1. Be as lean as possible without becoming underweight.
2. Be physically active for at least 30 minutes every day.
3. Avoid sugary drinks. Limit consumption of energy-dense foods (particularly processed foods high in added sugar, low in fiber, or high in fat).
4. Eat a variety of vegetables, fruits, whole grains, and legumes such as beans.
5. Limit consumption of red meats (such as beef, pork, and lamb), and avoid processed meats.
6. Limit alcoholic drinks to 2 a day for men and 1 a day for women.
7. Limit consumption of salty foods and sodium-processed foods.
8. Don't use supplements to protect against cancer.
9. Mothers should breastfeed exclusively for up to 6 months before adding other liquids and foods.
10. After treatment, cancer survivors should follow these recommendations for cancer prevention.

